# Supplementary material for: Pre-Hospital Pain Management in Children with Injuries: A Retrospective Cohort Study
Source: J Clin Med. 2021 Jul 9;10(14):3056. doi: 10.3390/jcm10143056 (PMC8307009; doi:10.3390/jcm10143056)
Supplement: Supplementary file 1 [file jcm-10-03056-s001.zip › jcm-1259082-supplementary.pdf]

Article

Pre-hospital pain management in children with injuries: A retrospective cohort study.

Ada Holak, Michał Czapla and Marzena Zielińska

Supplementary

Table S1. **A comparative analysis of the treatment of the same types of injuries, by age category**

		Age category										p-value*
		Infants		Toddlers		Preschool-age children		School-age children		Adolescents		
		n	%	n	%	n	%	n	%	n	%	
**Sex**	Males	49	53	165	61	187	67	525	67	627	61	**p<0,014**
	Females	43	47	107	39	91	33	261	33	395	39	
**Injury**	Low-energy	37	40	144	53	181	65	560	71	684	67	**p<0.001**
	High-energy	51	55	72	26	83	30	221	28	330	32	
	Burn-related	4	4	57	21	14	5	6	1	8	1	
**Accident site**	home	67	73	218	80	144	52	213	27	196	19	**p<0.001**
	school	6	7	14	5	44	16	293	37	342	33	
	street and road traffic	12	13	17	6	37	13	126	16	218	21	
	agriculture	1	1	-	-	-	-	3	0	3	0	
	public place	6	7	24	9	53	19	148	19	261	26	
	work	-	-	-	-	-	-	-	-	2	0	

***χ^2 test, n—number of patients; %—percentage of patients;**

Table S2. An analysis of the frequency of immobilisation and/or cooling by age group.

		Age category										p-value*
		Infants		Toddlers		Preschool-age children		School-age children		Adolescents		
		n	%	n	%	n	%	n	%	n	%	
**Cooling**	No	88	96	239	88	263	95	780	99	1,020	100	**p<0.001**
	Yes	4	4	34	12	15	5	7	1	2	0	

**Immobilisation**	No	89	97	256	94	240	86	601	76	728	71	**p<0.001**
	Yes	3	3	17	6	38	14	186	24	294	29	

*χ^2 test; n—number of patients; %—percentage of patients;

Table S3. An analysis of frequency of use of individual analgesics and sedatives in designated age categories.

		**Age category**										
		Infants		Toddlers		Preschool-age children		School-age children		Adolescents		**p-value***
		n	%	n	%	n	%	n	%	n	%	
**Acetaminophen**	No	90	98	249	91	267	96	750	95	989	97	**p=0.002**
	Yes	2	2	24	9	11	4	37	5	33	3	
**Ibuprofen**	No	92	100	270	99	277	100	784	100	1,017	100	p=0.59
	Yes	-	-	3	1	1	0	3	0	5	0	
**Acetylsalicylic acid**	No	92	100	273	100	278	100	787	100	1,022	100	p=1.00
	Yes	-	-	-	-	-	-	-	-	-	-	
**Ketoprofen**	No	92	100	273	100	278	100	779	99	962	94	**p<0.001**
	Yes	-	-	-	-	-	-	8	1	60	6	
**Metamizole**	No	92	100	273	100	278	100	783	99	1,001	98	**p=0.001**
	Yes	-	-	-	-	-	-	4	1	21	2	
**Fentanyl**	No	91	99	273	100	275	99	779	99	988	97	**p<0.001**
	Yes	1	1	-	-	3	1	8	1	34	3	
**Morphine**	No	88	96	246	90	260	94	734	93	963	94	p=0.15
	Yes	4	4	27	10	18	6	53	7	59	6	
**Midanium**	No	92	100	271	99	275	99	787	100	1,015	99	p=0.11
	Yes	-	-	2	1	3	1	-	-	7	1	
**Diazepam**	No	92	100	267	98	275	99	786	100	1,018	100	**p=0.001**
	Yes	-	-	6	2	3	1	1	0	4	0	

*χ^2 test; n—number of patients; %—percentage of patients;
